# 吉非替尼治疗晚期非小细胞肺癌疗效的相关分子生物学因素研究进展

**DOI:** 10.3779/j.issn.1009-3419.2010.03.10

**Published:** 2010-03-20

**Affiliations:** 116011 大连，大连医科大学附属第一医院肿瘤内科 First Affiliated Hospital of Dalian Medical University, Dalian 116011, China

非小细胞肺癌（non-small cell lung caner, NSCLC）占肺癌的80%，确诊时多为晚期。虽然手术、化疗、放疗技术在不断提高，但NSCLC患者的预后仍然很差，总体5年生存率仍然小于20%^[1]^。近年来一些针对肿瘤特定分子靶点的抗肿瘤靶向药物显示了一些疗效，其中以表皮生长因子受体（epidermal growth factor receptor, EGFR）为靶点的EGFR酪氨酸激酶抑制剂（EGFR tyrosine kinasesinhibitor, EGFR-TKI）吉非替尼（Gefitinib）在NSCLC的治疗中显示了一定的疗效。吉非替尼是一种低分子量的苯胺喹钠唑啉化合物，IDEAL1和IDEAL2的Ⅱ期试验结果显示其治疗晚期NSCLC有效^[2]^，且副反应易于耐受^[3]^。临床回顾性研究显示吉非替尼对不吸烟者、亚裔、女性、支气管肺泡癌或腺癌伴支气管肺泡癌分化患者有效。近年来研究发现EGFR基因突变等分子生物学因素与吉非替尼的疗效相关。本文就近年来与吉非替尼治疗晚期NSCLC疗效相关的分子生物学因素做一概述。

## 1 *EGFR*基因

### 1.1 *EGFR*基因突变

EGFR是一种蛋白酪氨酸激酶受体，它含有一个胞外配体结合结构域、一个跨膜结构域和一个具有酪氨酸激酶活性的胞浆结构域。其胞外部分与EGF或转化生长因子α（transforming growth factor α, TCF-α）等结合后，引起EGFR同源二聚化或与其他相关受体（如neu）异源二聚化而活化它们的胞浆位点，使受体酪氨酸磷酸化，进而活化第二信使。主要有3条信号传导通路：通过MAPK途径诱导细胞外信号的活化、通过PI3K途径活化AKT和通过JAK活化信号转导子和STAT的转录活化子，这些都是EGFR所介导的生长作用或致癌的基本下游途径。异常的EGFR活化机制至少包括基因拷贝数的增加、蛋白的过度表达和基因突变，从而导致肿瘤的产生^[4]^。

*EGFR*基因位于人类7号染色体的短臂7p12-14区，包括28个外显子。外显子18-21是*EGFR*基因的酪氨酸激酶的ATP结合位点的编码区，与吉非替尼治疗相关的EGFR突变多在于此处^[5]^。最常见的突变包括外显子19区的缺失突变和外显子21区的点突变，这两种突变约占所有EGFR突变的85%-90%。外显子19区的碱基缺失主要是第746-752位密码子的缺失突变，分别编码亮氨酸、精氨酸、谷氨酸、丙氨酸，导致EGFR蛋白中氨基酸序列丢失，这一缺失改变了受体酪氨酸激酶ATP结合槽（ATP-binding cleft）的角度，从而改变了细胞对EGFRTKIs的敏感性。外显子21的点突变主要是第858位密码子出现T→G的转换，引起EGFR蛋白中该位点的氨基酸由亮氨酸变为精氨酸（CTG→CGG, L858R）^[6]^，另外还可发生第861位的亮氨酸被谷氨酸替代（L861Q）。外显子18、20突变少见，外显子18多见碱基置换突变，包括G719C、G719A、G719D；外显子20发生突变类型较多，包括点突变、插入突变、碱基置换突变，还包括S768I、V819A、T790M等，其中点突变未发现会引起氨基酸序列的改变^[5]^。

2004年Lynch等^[7]^对16例服用吉非替尼治疗的肺癌患者的*EGFR*基因测序发现，9例治疗有效的患者中8例存在基因突变，而7例无效的患者均为野生型，首次揭示*EGFR*基因突变与药物的疗效有关，同时发现带有EGFR突变的细胞对EGF的反应性提高，对吉非替尼的敏感性也增加，认为EGFR变异使得酪氨酸激酶ATP结合位点的关键基团发生重构，增强了EGFR与ATP或其竞争性抑制剂的相互作用。许建明等^[8]^对106个口服吉非替尼的病人的标本进行检测显示，EGFR突变率为30.2%，中位疾病进展时间（time to progression, TTP）和总生存期（overay survival, OS）突变型比野生型明显延长，分别为15个月*vs* 3个月（*P* < 0.000 1）、18.5个月*vs* 6个月（*P* < 0.000 1）。这些发现也相继被许多其他国家的研究人员所证实，并发现EGFR突变和患者的临床病理特征有明显的相关性，如非吸烟、女性、亚裔、腺癌尤其是细支气管肺泡癌的NSCLC患者中EGFR的突变率较高。这些结果与种族、病理类型、吸烟及性别与患者对TKIs的疗效有关恰好相符^[6]^。Chan等^[9]^总结了15个研究中近3 000例NSCLC患者的EGFR突变，发现在不吸烟患者中突变率（34.8%）明显高于吸烟者（7.8%），女性突变率（26.4%）高于男性（9.3%），东亚人群突变率（30.6%）高于高加索人群（7.6%），在腺癌中的突变率为23.2%，肺泡细胞癌为17.9%，远高于其他组织类型（2.2%）。朱建权等^[10]^还发现在肺癌患者中EGFR外显子19突变比21突变对吉非替尼有更好的有效率。近期的IPASS研究^[11]^显示在EGFR突变阳性的患者中无进展生存期（progression-free survival time, PFS）在吉非替尼组明显长于卡铂/紫杉醇组（OR=0.48, *P* < 0.001），客观缓解率为71.2% *vs* 47.3%（*P* < 0.001）。目前许多的研究已经证实伴有EGFR突变的患者有更好的吉非替尼获益率，*EGFR*基因突变与吉非替尼的疗效相关，是预测吉非替尼疗效的最佳指标。

### 1.2 *EGFR*基因拷贝数扩增

近年来的研究结果显示*EGFR*基因拷贝数的扩增与TKI的疗效有相关性。Han等^[12]^采用*FISH*方法对66例口服吉非替尼的肺癌患者进行检测，发现高*EGFR*基因拷贝数的患者有更好的疗效，同时发现高*EGFR*基因拷贝数与EGFR突变密切相关。虽然观察到高*EGFR*基因拷贝数的患者有较长的TTP和OS，但是没有统计学意义。John等^[13]^应用*CISH*方法对36例口服吉非替尼的NSCLC患者进行检测，发现*EGFR*基因扩增与EGFR突变密切相关，提示*EGFR*基因扩增也是吉非替尼的敏感预测指标。

### 1.3 *EGFR*基因第1内含子区CA-SSR重复序列的多态性

*CA-SSR*基因多态性作为一种最常见的遗传变异形式，近年来已经成为药物疗效相关性研究的热点。多项研究^[14]^表明*EGFR*基因第1内含子区CA-SSR多态性能影响EGFR活性，并在体外影响EGFR-TKI对肿瘤细胞的抑制活性。近年马飞等^[14]^对80例晚期NSCLC患者进行的单药吉非替尼临床实验表明，携带短CA重复序列患者的客观有效率和临床获益率均明显高于长CA重复序列的患者。Nie等^[15]^也进行了相关实验，结果与马飞等观察的结果相似，但在远期生存分析中发现短CA重复序列的患者采用吉非替尼治疗后存活时间明显长于长CA重复序列的患者，推测其分子机制为CA-SSR多态性可影响EGFR的mRNA和蛋白表达水平，从而影响EGFR信息传导通路的活性。在多种肿瘤中已经证实CA-SSR为不良预后因素，携带该基因型的肿瘤患者生存期更短^[16]^。由于参与实验的患者样本量小，所以目前还很难从理论上阐明携带短CA重复序列的患者服用吉非替尼的远期生存情况，还需要开展更大样本的前瞻性临床研究来证实CA-SSR与吉非替尼疗效的相关性。

### 1.4 *EGFR*基因突变的耐药机制

并不是所有的EGFR突变都会导致吉非替尼的敏感性增加，对吉非替尼最初有反应的患者可发生二次突变而出现吉非替尼的耐药，如T790M突变。T790M突变导致大量的甲硫氨酸残基与苏氨酸在EGFR激酶区基因790位置的置换。Engelman等^[17]^所做的检测T790M突变的发生是否能引起对吉非替尼敏感的细胞系抗药性的研究表明，外源性T790M的引入能对吉非替尼产生抗药性。近年国内外很多研究^[18, 19]^显示，接受吉非替尼治疗的患者中，T790M突变率高于未接受吉非替尼治疗的患者，提示T790M突变可能与吉非替尼诱导作用有关；有些患者在治疗前即有T790M突变且与较差的PFS有关，更多的患者无T790M突变却对吉非替尼不敏感，提示还存在其他未知的耐药机制。

## 2 EGFR蛋白表达

研究^[20]^显示EGFR蛋白过度表达与吉非替尼疗效有相关性。Hirsch等^[21]^采用免疫组化方法检测NSCLC患者的EGFR蛋白表达情况，发现阳性患者比阴性患者有更长的总生存期。但Cappuzzo等^[22]^对43例标本进行免疫组化检测未证实EGFR蛋白表达水平可以预测肿瘤对吉非替尼治疗疗效的敏感性。Villaflor等^[23]^也应用免疫组化的方法检测吉非替尼EAP研究中87例标本的EGFR蛋白，同样认为EGFR蛋白表达不能预测患者的预后。美国科罗拉多州大学癌症中心的研究^[24]^发现EGFR蛋白的高表达与较好的治疗效果有关，但进一步的统计分析表明EGFR蛋白高表达的患者与无EGFR蛋白高表达的患者相比疗效无明显差异。EGFR蛋白表达预测吉非替尼疗效的意义还需要去验证。

## 3 人类表皮生长因子受体2（human epidermal growth fac- tor receptor, Her-2）突变

Her-2是EGFR家族的又一成员，又名erbB2，可与EGFR形成异源二聚体，激活EGFR的酪氨酸激酶区域的自身磷酸化，从而激活下游信号通路，促进肿瘤细胞的生长、增殖。这种异源二聚体与EGFR同源二聚体相比有更高的稳定性、再利用率和信号传导能力。Han等^[12]^对57例可评价的患者进行直接测序，发现有4例患者含有Her-2突变，但是均对吉非替尼无反应。Her-2突变通常是外显子20发生删除或复制，多发生在不吸烟和肺腺癌的患者。近年也有对Her-2突变与EGFR-TKI临床疗效的相关报道。研究^[25, 26]^发现有Her-2突变的NSCLC患者对EGFR- TKI产生耐药性，但仍对Her-2靶向治疗药物敏感，除去突变的Her-2后，癌细胞可恢复对EGFR-TKI的敏感性，这表明Her-2突变可能导致EGFR-TKI产生耐药性。

## 4 磷酸化AKT（phosphorylated, p-AKT）

AKT是一种丝氨酸/苏氨酸蛋白激酶，与蛋白激酶A（protein kinase A, PKA）、蛋白激酶C（protein kinase C, PKC）有高度的同源性，又名蛋白激酶B（protein kinase B, PKB），是体内癌细胞生存通路PI3K/AKT信号通路的重要的信号转导蛋白，并处于此信号通路的枢纽位置，在细胞的存活机制和介导肿瘤发生的信号转导中有重要作用。而多种生长因子、激素、细胞因子、癌基因（RAS）、磷酸酶和细胞骨架张力蛋白同源物（phospha- tase and tensin homolog, PTEN）等均可经PI3K的信号传导作用使AKT催化结构域的Thr308位点和调节结构域的Ser473位点发生磷酸化，从而使AKT活化，只有磷酸化的AKT才具有生物学特性^[27, 28]^。

Cappuzzo等^[27]^发现女性、不吸烟和具有细支气管肺泡癌特征的接受吉非替尼治疗的晚期患者中，p-AKT蛋白阳性表达率增高，并且p-AKT阳性与p-AKT阴性患者在吉非替尼治疗反应率、疾病控制率以及TTP的比较中更有优势，比值分别是26.1% *vs* 3.9%、60.9% *vs* 23.5%及5.5个月*vs* 2.8个月。因此推测p-AKT表达水平高的NSCLC患者对吉非替尼的反应性较好^[28]^。

## 5 核苷酸切除修复交叉互补基因1（excision repair cross- complementing 1, ERCC1）、核苷酸还原酶M1（ribo- nucleotide reductase M1, RRM1）

*ERCC1*定位于人类染色体19q13.2-19q13.3，大小为33 kb，是NER（nucleotide excision repair，核苷酸切除修复）途径活性的标志性基因，也是细胞存活必须的DNA修复基因，其编码产物是297个氨基酸。ERCC1在NER修复过程中发挥重要作用，它与XPF/ERCC2形成异源二聚体，具有5’核酸内切酶活性，可以在损伤位点15-24个核苷酸处切除DNA单链，进而参与DNA链的切割和损伤识别^[29]^。一般认为晚期患者ERCC1高表达往往导致癌细胞对铂类耐药，从而导致化疗失败^[30]^。RRM1是核苷酸还原酶的亚结构，核糖核苷酸还原成脱氧核糖核苷酸是DNA合成的重要步骤，核苷酸还原酶使5’-二磷酸核糖核苷酸转化成5’-二磷酸脱氧核糖核苷酸。

宋启斌等^[31]^仔细分析了ERCC1和RR M1的功能，指出ERCC1和RRM1在NSCLC不同的阶段具有不同的角色。在肿瘤早期通过预防突变，RRM1和ERCC1高表达可减少癌症的发生，或阻止已经存在的肿瘤的进展，所以对于未进行化疗的早期NSCLC患者，RRM1和ERCC1高表达是预后良好的标志，而对于晚期NSCLC，若进行化疗RRM1和ERCC1高表达则可能导致耐药的产生。因此，ERCC1和RRM1对于NSCLC患者治疗的选择存在两种情况：早期患者ERCC1和RRM1高表达则可以不进行化疗，而晚期患者ERCC1和RRM1高表达，则不适合传统的化疗，可使用EGFR抑制剂如吉非替尼。

## 6 *K-ras*基因突变

K-ras是小分子G蛋白，是EGFR通路的下游信号分子，在细胞的生长、分化等过程中起重要作用。*K-ras*基因是第一个被克隆分离的人类癌基因，有3种类型：*H-ras*、*N-ras*和*K-ras*。*K-ras*基因突变主要集中在第12位氨基酸残基，少数病例中也有第13位及第61位点突变。K-ras突变常见于吸烟、男性、腺癌患者^[12]^。Pao等^[32]^最先报道了含有K-ras突变的NSCLC患者对吉非替尼耐药。Akiko等^[33]^应用体外细胞实验检测转染K-ras 12V突变体的HEK293T细胞对吉非替尼的反应性，发现在高浓度吉非替尼的抑制下，细胞仍然大量增殖，提示含*K-ras*基因突变的患者对吉非替尼反应差，与不良预后有关。

## 7 表皮膜蛋白1（epithelial membrane protein-1, EMP-1）表达

EMP-1于1995年由Taylor V首次发现并命名，又称为（progression associated protein, PAP）、CL-20、TMP和B4B，与PMP22外周鞘磷脂蛋白同属一个跨膜家族，其全长含有157个氨基酸，有4个疏水跨膜螺旋区，染色体定位于12p12.3，根据氨基酸结构推测其定位在细胞膜上。

Jain等^[34]^检测了39例接受吉非替尼治疗的患者的肿瘤组织标本，发现吉非替尼治疗有效的患者的标本中无EMP-1的表达。在14例（28%）治疗评价为稳定或进展的患者中存在EMP-1的表达，更重要的是有1例患者开始时对吉非替尼敏感并且没有EMP-1的表达，但后来产生继发性耐药，当肿瘤复发时EMP-l出现明显表达，表明EMP-1是预测病人是否对吉非替尼耐药的敏感预测指标。在研究中还发现EMP-1的表达及吉非替尼的继发性耐药与EGFR的体细胞突变无关，该研究也为预测吉非替尼治疗有效性的研究提供了新的思路。宋启斌等^[31]^分析吉非替尼获得性耐药的患者发现，这些患者在诊断时为支气管肺泡癌，开始时对吉非替尼存在反应，由于存在L858R突变，吉非替尼治疗前的标本EMP-1表达很低（< 10%），这些患者治疗160天后出现耐药，分析标本发现出现了新的T790M突变，同时EMP-1表达的表达也明显上升，提示EMP-1是吉非替尼原发和获得耐药的生物标志，而与EGFR的激酶区域突变无关。

## 8 髓细胞瘤原癌基因（myelocy tomatosis oncogene, MYC）、真核翻译起始因子3（eukaryotic translation ini- tiation factor 3, EIF3）

分子细胞遗传学分析表明染色体长臂8（8q）的扩增在许多恶性疾病中经常发生，包括肺癌。MYC定位在8q24.1，是个很有特征的致癌基因，它涉及细胞的生长、分化和程序性细胞死亡。以前的研究^[35]^报道MYC扩增与肿瘤差的预后相关。MYC在肺癌细胞系中是最常扩增的基因。EIF3是一种巨大的多蛋白复合体，它被认为是结合40s核糖体亚单位并且阻止与60s亚单位结合的一种因子。EIF3包括EIF3A-EIF3M 13个亚单位。各种EIF3亚单位已经被发现在恶性的肿瘤中有表达。在人类，EIF3H已经被发现在前列腺癌、乳腺癌中过表达^[36]^。在许多的病例中，这可能归因于8q23区域的扩增，这个区域在8q23.3位点含有*EIF3H*基因。

Cappuzzo等^[37]^对54例NSCLC患者的*EIF3H*和*MYC*基因扩增情况进行FISH检测，EIF3H扩增在10例患者中被检测到，这10例患者同时存在MYC扩增，还有2例患者仅存在MYC扩增，所有的EIF3H FISH阳性的患者MYC也是阳性，统计分析表明MYC阳性的患者对吉非替尼有更高的反应率（*P*=0.003），更长的TTP（*P*=0.01）和OS（*P*=0.002）。在EIF3H阳性的患者中也有同样的发现，结果表明高的*EIF3H*和*MYC*基因拷贝数与EGFR-TKT的敏感性相关。但是由于此研究病例数少，这两个预测指标对吉非替尼疗效的预测性还需要进一步的研究。

## 9 层粘连蛋白5（Laminin5, LN5）

LN5是一个由一个长臂和三个短臂的构成的十字型结构。长臂的螺旋结构由三个链间形成二硫键共价组成（α3，β3和γ2）。短臂上直杆样的区域由以球型区域插入的类似表皮生长因子的基因重复组成。由于LN5的多区域结构，除了整联蛋白以外的其他的受体很可能用它的许多潜在的配体中的一个来调停不同细胞的作用，包括EGFR信号通路。因此很容易理解吉非替尼抑制EGFR表达，而高LN5表达激活了EGFR，并且抵制了吉非替尼的影响^[38]^。

LN5是细胞外基质蛋白，在细胞移行和肿瘤侵袭方面有重要作用^[39]^。安社娟等^[38]^实验结果提示高LN5 mRNA表达水平与肺癌患者应用吉非替尼的不良预后有关。LN5与吉非替尼之间的相互作用分子机制现在仍不清楚。在体外做的肝细胞癌（HCC）细胞株表明吉非替尼抑制肝细胞癌的生长，然而有剂量依赖性的LN5抑制了吉非替尼对EGFR下游信号通路的重要效应因子AKT的放射作用^[40]^。对EGFR信号通路的研究^[38]^也证实了这一点，与吉非替尼能抑制p-AKT相比，LN5与吉非替尼的复合体没有太多改变。他们的实验结果显示低LN5 mRNA水平患者中疾病控制率为52.9%，高表达的患者中疾病控制率为17.69%，证明体外实验外源LN5能中和吉非替尼对p-AKT的抑制作用，低LN5 mRNA水平的患者会获得更好的吉非替尼治疗疗效，提示LN5可能做为一个新的靶点单独或与其他靶点一起应用于肺癌患者的治疗。

## 10 *C-fos*基因

*C-fos*是编码核蛋白的癌基因，属于立早基因（early response genes, ERG）的成员，此类基因受到多种刺激后立即表达，产生转录因子，调控其他基因的转录及表达，调节正常细胞的生长、发育和分化。*Fos*基因蛋白表达产物定位于细胞核，目前已知有4种Fos蛋白，即C-fos、Fos B、Fra-1和Fra-2。*C-fos*与*C-jun*基因以亮氨酸拉链结构的形式形成同源或异源二聚体，这种同源或异源二聚体具有转录激活活性，故又称转录因子（Ap-1），以C-jun/C-fos二聚体形式的Ap-1生物学活性最强。作为转录因子的Ap-1与DNA结合后，可在转录水平调节下游靶基因的表达，参与细胞增殖及转化。研究表明Ap-1与细胞增殖相关，在肿瘤转化乃至逆转中均起关键作用。

Jimeno等^[41]^的体内外实验显示吉非替尼可以有效降低*C-fos*基因mRNA的表达水平，从而抑制肿瘤细胞的生长。*C-fos*基因的变化是机体对EGFR抑制剂产生作用的早期反应之一，可较早监测吉非替尼的药物作用。

## 11 胰岛素样生长因子1（insulin-like grow th factor, IGFR-1）

IGFR-1是一个EGFR信号传导旁路上的激酶受体，其与EGFR-TKI形成异源二聚体，激活IGFR-1，从而启动PI3K/AKT通路，促进生长素蛋白和EGFR重新合成，使得癌细胞增殖形成耐药性。当IGFR-1过表达时，就可能直接激活EGFR下游信号通路，促进癌细胞增殖。Cap- puzzo等^[42]^对124例口服吉非替尼的晚期NSCLC患者的病理组织进行免疫组化研究，未发现IGFR-1的表达与吉非替尼的耐药性有关，这可能与不同的实验方法有关。因此还需要大量的研究去进一步证明IGFR-1与吉非替尼耐药性之间的相关性。

## 12 乳酸脱氢酶（lactic dehydrogenase, LDH）

LDH是与无氧代谢和糖酵解密切有关的一组同工酶，有5个亚型LDH1、LDH2、LDH3、LDH4和LDH5，其中LDH5是与肿瘤的无氧代谢直接相关的一型同工酶^[43]^。在无心、肝、肾疾病及其它创伤的恶性肿瘤病人中，血清总LDH升高主要取决于LDH5的升高。Konjevic等^[44]^认为血清LDH水平是恶性肿瘤的预后因素。

施瑞浩等^[45]^对58例服用吉非替尼4周以上的无原发心脏、肝脏疾病及损伤的以腺癌、女性、不吸烟为主的晚期或复发NSCLC患者用药前后LDH水平做了分析，结果为控制组治疗后LDH水平有明显降低（*P* < 0.001）；未控制组患者治疗后LDH水平有明显升高（*P* < 0.001），表明血清LDH可协助判断吉非替尼在无原发心脏、肝脏疾病或损伤的选择性中晚期NSCLC中的疗效。但是由于对LDH与吉非替尼疗效的相关性的报道很少，还需要进一步验证。

## 13 外周血的相关标志物

Okamoto等^[46]^研究发现血清中的癌胚抗原（CEA）水平增高可以作为吉非替尼有效率的预测指标，它同样也是晚期NSCLC病人接受这一治疗的预后因素。Ishi- kawa等^[47]^研究发现血清中的双调蛋白及转化生长因子-α（TGF-α）能够作为晚期NSCLC病人对吉非替尼耐药的重要的预测因子，两者阳性表达标志着对吉非替尼的反应及预后好，且两者在男性病人、非腺癌病人及吸烟者中阳性率更高，这为寻找有效的预测指标提供了全新的思路，即在外周血中寻找预测指标，但仍需进一步的研究加以证实^[48]^。

## 14 问题与展望

吉非替尼是最先被批准上市的小分子靶向治疗药物，其对部分NSCLC患者疗效明显。目前比较确切的与疗效相关的分子生物学因素是EGFR突变。应该看到NSCLC增殖及转移是多靶点多环节的调控过程，这就可能需要应用多种分子生物学方法，对多种基因、蛋白表达进行分析。单一因素来预测疗效也许是不全面的，需要多因素检测分析。通过分子生物学因素分析，结合患者的临床特点，找到吉非替尼敏感或耐药的分子水平预测因素，真正实现NSCLC的个体化靶向治疗。另外，各种检测手段的规范化也是需要及早解决的问题，快速、规范的检测是实现疗效预测的重要保证。
